# A Case of Excessive Masturbation in the Female, with an Unusual Method of Performance

**Published:** 1882-09

**Authors:** J. H. Pryor

**Affiliations:** Resident Physician Buffalo General Hospital


### Clinical Reports.

### A Case of Excessive Masturbation in the Female, with an Unusual Method of Performance.

Reported by J. H. Pryor, M. D.,

Resident Physician Buffalo General Hospital.

Miss K. T., æt. 32, single, domestic, Scotland, entered hospital July 16, 1882, accompanied by Dr. Hopkins, of Silver Creek, who gave the following history: Patient is a chronic masturbator who has kept up the practice for years without her relatives' knowledge. Three days ago, in a fit of despondency caused by the fear that she had permanently ruined her health and shattered her mind by the excessive indulgence of the day before, she procured a knife and endeavored to end her existence. The doctor was summoned, and the truth became known. It was thought advisable to gain admission for her into some institution where she could be watched and her mind directed to other matters. Her presence is typical: she has the pimply, nervous face; the downcast, furtive glance; the jerking, nervous motion: an air of one deeply lost in unpleasant thought, with a cringing, fearful manner; is very talkative, but speaks only of herself; while in conversation her gaze is directed to her hands, which she seems to be examining closely; blushes easily. On referring to her habit, she weeps and gives a shrug of horror. Her language is devoid of all obscenity. She seems not to be depraved or incestuous, but talks like one in deep grief and humiliation; has dyspepsia with a ravenous appetite; insomnia, cephalalgia, deafness at times, constipation, dry mouth and tongue, stammering badly at times, and occasional cardiac palpitation; is also very anæmic; menstruates regularly. When pressed for a complete history, she stated that when five years old she used to amuse herself by a peculiar swaying of her person, each time experiencing delight and pleasure from its performance. For this reason she became addicted to this habit. Her parents noticed it, but thinking it a childish freak, did not reprimand her nor speak of its baneful influence. Thus she continued first for pure enjoyment, then as a solace when in trouble or sorrow. "When things went wrong this furnished the needed relief from worry."

Again she would be very excitable, her head would throb and ache, her mind would be a babel of thought; an indulgence would leave its sedative influence to calm her. Finally this act became essential to preserve her nervous equilibrium. By this method she could make her morbid thoughts and feelings something akin to those of other folks. It enabled her to depress and control a heightened and rosy imagination, and to free herself from the grasp of a melancholy and dejected mood. At first she would defile herself but once a day, but later she became as much a slave to it as the opium eater to his drug.

If a simple task devolved upon her, she must needs resort to this act to gain the composure and steadiness necessary for its performance, and it alone ensured a sleep full of bright and radiant dreams. Of late years trouble has been the principal incentive to pollution, in her serener moments the thought of her self-abasement and wrecked life driving her to desperation. At such times her masturbation became painfully excessive, having frequently spent hours in wild enjoyment, which was so nearly allied to pain, that it leads me to think that it must have been associated with her mental delirium. Now there came a lull when she suffered. For four long months she endeavored to be a woman, but something tripped up her resolve and she fell away down into the depth. This marked the commencement of a period of groveling and despair, which ended on the morning of her suicidal intent. When I asked her in regard to her method of pursuing this habit, she said that she could show me better than she could tell me, and straightway falling upon her knees before a chair, she let her elbows drop upon its seat and grasping the arms with a firm grip she commenced a swaying, writhing motion, seeming to fix her pelvis and moving her trunk and limbs,—this for a moment—then every muscle was rigid, in contraction, her face borrowed a demoniac expression; the features were contorted; the eyes rolled; the teeth set and lips compressed, while the cheeks wore a purplish flush. Thus she remained for a time a most revolting spectacle. Upon rising she explained "that it only took a moment to commence, but that the reveling lasted for a long time. She then showed me her knees which bore the marks of her yesterday's debauch, being skinned and swollen with some old ecchymosis, indications of times gone by; also remarking that often her muscles were so lame the day after an indulgence that she could scarcely lift her hand. Not being able to understand how the orgasm could be thus produced, I took occasion, a day or two later, to question her explicitly in regard to this point. She answered that the swaying induced a pleasurable sensation, which was probably caused by suction being brought to act upon the clitoris. This suction she describes distinctly, and says that almost immediately after something bursts, and then comes the rapture which is prolonged for an indefinite time. Probably the sensation of something bursting is caused by the discharge from the vulvo-vaginal glands. I am at a loss to explain the greatly prolonged orgasm which she describes; whether it be a psychical delusion or a physical phenomenon I do not know, but it seems eminently proper that I should state here that if it be a delusion I have never discovered another. She has tried other methods of masturbation, but finds them very unsatisfactory. An examination revealed a slightly enlarged clitoris slightly congested and tender; vagina somewhat tender; uterus normal. The patient was placed in the ward under observation; given some work, also baths, iron and light doses of pot. brom. If she chanced to speak of the irreparable injury she had done herself, a firm assurance was given her that this was not true and that she would certainly and surely get well. To-day, Aug. 14, the improvement is very marked; she has gained in flesh; is more cheerful; complains very little of headache, and believes that she will recover. So far as can be learned she has not resorted to her old practice. Whether her recovery will be permanent is a question, as she probably will not remain here a sufficient length of time. I have searched the literature as far as I am able to, and fail to find a similar case recorded. I therefore present it more for its oddity and strangeness than for any practical thoughts or knowledge which it affords or suggests.

